# Role of Toll-like Receptors Nine and Ten Polymorphisms in Childhood Bronchial Asthma Control and Their Relation to Cardiac Function

**DOI:** 10.3390/diagnostics15070817

**Published:** 2025-03-24

**Authors:** Rehab Ahmed Rabie, Asmaa Elsharkawy Hussien, Hesham Samy Abdelhameed, Soad Abdelsalam Shedeed, Noura Almadani, Hanaa A. Nofal, Dina S. El-Rafey, Hossam T. Ali, Mohammed Sanad Naguib

**Affiliations:** 1 Medical Microbiology and Immunology Department, Faculty of Medicine, Zagazig University, Zagazig 44519, Egypt; dr.rehabrabie@yahoo.com; 2 Pediatrics Department, Faculty of Medicine, Zagazig University, Zagazig 44519, Egypt; a.alsharqawi23@medicine.zu.edu.eg (A.E.H.); hsa.noureldin@gmail.com (H.S.A.); soadshedeed@gmail.com (S.A.S.); mohammedsanad37@yahoo.com (M.S.N.); 3 Community and Psychiatric Mental Health Nursing Department, College of Nursing, Princess Nourah bint Abdulrahman University, P.O. Box 84428, Riyadh 11671, Saudi Arabia; naalmadani@pnu.edu.sa; 4 Community, Environmental Occupational Medicine Department, Faculty of Medicine, Zagazig University, Zagazig 44519, Egypt; dselrafey@gmail.com; 5 Qena Faculty of Medicine, South Valley University, Qena 83523, Egypt; hossamtharwatali@gmail.com

**Keywords:** toll-like receptors, childhood, bronchial asthma, cardiac function

## Abstract

**Background:** Asthma is designated as the most widely spread chronic disease in children. Toll-like receptors (TLRs) are linked to several human diseases, including allergic diseases. We aimed to assess the link between TLR9 (rs187084) and TLR10 (rs11096956) gene polymorphisms and bronchial asthma and its control and their relation to respiratory and cardiac functions. **Methods:** This is a case-control study comprising 80 participants aged between 5 and 12 years old, divided into 20 healthy non-asthmatic participants and 60 asthmatic ones. The asthmatic group members were diagnosed clinically according to the diagnosis guidelines of The Global Initiative for Asthma (GINA) 2019 and subdivided according to GINA 2019 guidelines for asthma control into three subgroups (well-controlled, partially controlled, and uncontrolled). Genetic polymorphisms in TLR9 (rs187084) and TLR10 (rs11096956) were detected using real-time PCR. **Results:** We found a significant increase in TLR9 polymorphisms among asthmatic cases compared to the control (OR = 9.09 for the CT genotype and 5.24 for the TT genotype) and a similar increase in TLR10 polymorphisms (OR = 4.29 for the GT genotype and 10.71 for the TT genotype). Also, there was a significant increase in TLR9 and TLR10 polymorphisms among uncontrolled cases compared to both well-controlled cases and the control group. We discovered a significant association between TLR9 (rs187084) gene polymorphisms and pulmonary function tests (PFTs), with better results in the CC genotype. Additionally, a significant association with both RVFWSL (right ventricle free-wall longitudinal strain) and GLS (left ventricle global longitudinal strain apical 2-chamber view) with better values was linked to the CC genotype. Regarding TLR10 (rs11096956), there was a significant association between gene polymorphisms and PFTs, with better function in the GG genotype. Additionally, there was a significant association between TLR10 (rs11096956) gene polymorphisms and GLS AVG (left ventricle global longitudinal strain average), with the GG type having significantly better cardiac function. **Conclusion:** Subclinical cardiac dysfunction of the left and right ventricles was detected in asthmatic children. The CC genotype of TLR9 and the GG genotype of TLR10 are associated with better asthma control and better cardiac function. Therefore, TLR9 and TLR10 have a role in asthma control and cardiac dysfunction.

## 1. Introduction

The most common chronic illness affecting children is asthma, which puts persistent stress on the healthcare system. Recently, the occurrence of asthma symptoms in children and adolescents has increased worldwide, especially in low-middle income countries (LMICs). It places a heavy load on people and the community over the course of their lives [1]. It is a serious public health problem worldwide, which can influence the quality of life. Globally, asthma ranks 16th among the leading causes of years lived with disabilities and 28th among the primary causes of burden of disease, as assessed by disability-adjusted life years (DALYs) [2]. The global prevalence of BA in children ranges from 9.1% to 9.5%, rising to 10.4% in adolescents [3]; the prevalence of asthma and its rate of mortality in children have increased significantly over the past 40 years. The World Health Organization (WHO) estimates that approximately 300 million people worldwide suffer from asthma, and this figure is expected to reach 400 million by 2025 [4]. Globally, the mortality rate of pediatric asthma ranges from 0 to 0.7 per 100,000 people [5,6]. Among children, asthma is the most common chronic disease, ranking among the top 20 causes of DALYs worldwide in children of all ages [7].

The available information indicates that asthma is a multifaceted illness, and its development is becoming progressively more linked to an interplay of genetic susceptibility, environmental factors, and host factors [8]. Furthermore, a thorough understanding of the immunology of this condition can substantially help all asthmatics receive personalized, targeted treatment [9].

The initial line of defense against germs is made up of a family of receptors called toll-like receptors (TLRs). They are essential in bridging innate and adaptive immunity because they can detect both external pathogens and dangerous internal chemicals generated by injured or dying cells [10]. TLRs are widely distributed in the respiratory tract epithelium, where they help to activate immunological responses. Principally, the function of some TLRs during inflammation has raised the possibility that they have a role in the pathophysiology of asthma [11].

Several mechanisms can explain the impact of chronic lung diseases on the heart, including recurrent hypoxemia, the release of inflammatory mediators, changes in the pulmonary vasculature, and increased intra-thoracic pressure. These mechanisms directly affect the right ventricle by increasing the pulmonary artery pressure. Left ventricular dysfunction was also reported with chronic lung diseases, secondary to changes in the interaction between the right and left ventricles and changes in the ventricular preload and afterload [12].

Therefore, we intended to assess the relation between TLR9 and TLR10 gene polymorphisms and asthma and its control and the effect of asthma and gene polymorphisms on the respiratory and cardiac functions of the left and right ventricles using a new model of echocardiography (speckle tracking method) to detect subclinical cardiac dysfunction, if present.

## 2. Patients and Method

This case-control study was conducted at the Pulmonology Unit, Pediatric Department, Zagazig University Children’s Hospital in cooperation with the Echocardiography Unit, Pediatric Department, Zagazig University Children’s Hospital, and the Scientific and Medical Research Center of the Faculty of Medicine, Zagazig University, over a period of 35 months between December 2019 and October 2022.

### 2.1. Sample Size

The sample size was calculated using G power 3.1.9.7 based on the large expected effect size between the asthmatic group and control group for the frequency of TLR10 polymorphisms (d = 0.5), with CI 95%, power 80%, and an allocation ratio of 3:1. Therefore, the sample size was calculated to be at least 56 cases, with 14 in each group.

### 2.2. Participant Groups Involved in the Study

- The asthma group:

This study encompassed 60 asthmatic children. This group was divided into the following 3 subgroups according to the GINA 2019 guidelines [13] for asthma control:

1. Well-controlled group, including 20 children
2. Partially controlled group, including 20 children
3. Uncontrolled group, including 20 children

### 2.3. Inclusion Criteria

Aged between 5 and 12 years and diagnosed with bronchial asthma based on The Global Initiative for Asthma (GINA) diagnosis guidelines for 2019 [13].

### 2.4. Exclusion Criteria

Patients outside the age group, asthmatic patients with clinical impairment other than asthma (e.g., congenital heart diseases, congenital pulmonary diseases, chronic lung, heart, or kidney diseases, endocrinal or congenital or acquired immune deficiency diseases).

- The control group:

It involved twenty healthy children matched in age, sex, and BMI with the asthmatic case group to overcome any confounding factors; these participants were specifically chosen based on the absence of any indications of bronchial asthma, other pulmonary diseases, allergy, or atopy by clinical assessment and careful history taking. They were selected randomly from children coming to the pediatric outpatient clinic for other causes besides respiratory or cardiac symptoms (e.g., error of refraction, audiometric assessment).

### 2.5. Operational Design

All patients involved in the study underwent the following: **1—Demographic data,** such as socio-economic status, age, and sex. **2—Full medical history** focusing on the family history of asthma and other atopic diseases, when symptoms first appeared, the frequency of daytime symptoms, the frequency of awakening at night from exacerbations, the effect on normal activities, including school attendance, the types and route of drugs used, and previous hospital admissions. **3—Careful clinical examination**, including general examination, anthropometric measurements, local pulmonary examination, and other system examinations to rule out other chronic diseases. **4—Laboratory investigations**, including complete blood counting, liver function testing, kidney function testing, and C-reactive protein measuring. **5—Identification of exposure to TLR9 and TLR10 gene polymorphisms** [14] via real-time PCR. Each participant had two ml of venous blood drawn into sterile EDTA-containing tubes under strict aseptic conditions. To extract DNA, samples were kept at −20 °C or below. Following the manufacturer’s guidelines, the Gene JETTM whole blood genomic DNA purification mini kit (Thermo Scientific, Waltham MA, USA) was employed to extract DNA from the entire blood. The extracted DNA was then kept at −20 °C for a genotyping test [15]. The DNA concentration in each sample was assessed using a Nanodrop spectrophotometer (Thermo Scientific, USA). The real-time PCR thermal cycler (Applied Biosystems, Brooklyn, NY, USA) was used to identify the TLR9 (rs 187084 T/C) and TLR10 (rs11096956 T/G) SNPs. Using the TaqManTM genotyping master mix (Thermo Scientific, Waltham, MA, USA) and TaqMan Genotyping assays for TLR9 (rs 187084) and TLR10 (rs 11096956) (Thermo Scientific, Waltham, MA, USA), a total reaction volume of 20 μL was procured, encompassing 10 μL TaqManTM genotyping master mix, 1 μL SNP genotyping assay, and 20 ng genomic DNA diluted with DNA/RNA-free water to 9 μL. The following cycling conditions were used: 10 min polymerase activation at 95 °C, followed by 40 cycles that include a 15 s denaturation step at 95 °C and annealing/extension at 60 °C for 1 min.

### 2.6. Pulmonary Function Testing

Using the Jaeger Master Screen TM IOS, version 5.2, produced by VIASYS Healthcare GmbH, Hoechberg, Germany, under standard conditions and in accordance with the manufacturer’s instructions, pulmonary function testing was carried out at our pulmonology unit. Forced vital capacity (FVC) and forced expiratory volume in the first second (FEV1) were all tested using the standardized criteria [16].

### 2.7. Conventional Echocardiography and Two-Dimensional Speckle Tracking Analysis (Transthoracic)

To perform echocardiography, a 35-MHz phased array transducer (Philips EPIQ CVx, Amsterdam, The Netherlands) was utilized following the guidelines of the American Society of Echocardiography. Apical, subcostal, and parasternal views were obtained.

### 2.8. Study Outcome

Primary outcome: The role of toll-like receptors nine and ten polymorphisms in the occurrence of childhood bronchial asthma.

Secondary outcome: The role of toll-like receptors nine and ten polymorphisms in bronchial asthma control and their relation to respiratory and cardiac functions in bronchial asthma cases.

### 2.9. Statistical Analysis

The data were analyzed using Statistical Package for Social Science (SPSS), version 26, where qualitative data were presented as frequencies and percentages. For quantitative variables (e.g., mean, standard deviation (SD), and minimum–maximum), Chi square (χ^2^) with Yate’s correction and one-way ANOVA with post hoc Tukey test were employed. Also, Pearson’s correlation coefficient was determined to evaluate the relationship between various study variables. The results were considered statistically significant and highly statistically significant when the significant probability (*p*-value) was <0.05 and <0.001, respectively.

## 3. Results

**Figure 1:**
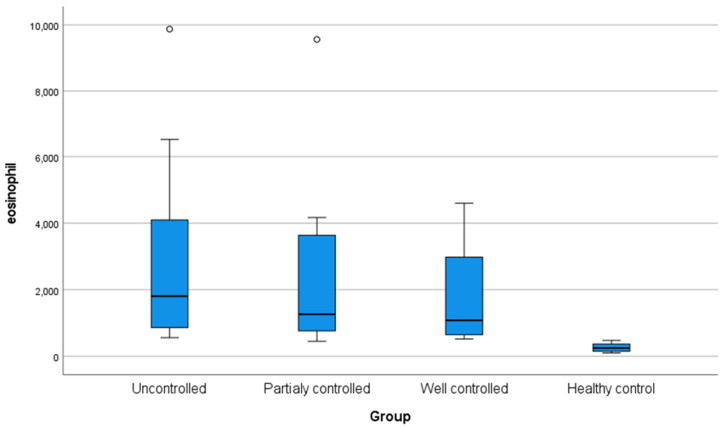
Box plot demonstrating eosinophil level (cells/μL) in the studied groups.

The current study showed no statistically significant differences in age, sex, weight, height, and body mass index among the four groups under investigation. However, the studied groups differed significantly in terms of family history, as 75% of the uncontrolled patients revealed a positive family history, whereas all control participants exhibited no positive family history (Table 1). The investigated groups revealed a significant statistical difference (*p* < 0.05) concerning eosinophilic count, with all asthmatic groups significantly elevated compared to the controls (Figure 1).

Table 2 shows that there was no statistically significant difference between the four studied groups regarding the ejection fraction (EF) (*p* = 0.41) and fractional shortening (FS) (*p* = 0.20). Conversely, there was a statistically significant difference between the four studied groups regarding the pulmonary artery systolic pressure (PASP) (*p* = 0.049), with the highest mean value reported in the uncontrolled asthma group, and regarding the tricuspid annular plane systolic excursion (TAPSE) (*p* = 0.002), with the highest mean values reported in the control group. In addition, there was a statistically significant difference between the four studied groups regarding the right ventricular echocardiographic measurements, such as right ventricle free-wall longitudinal strain (RVFWSL) (*p* < 0.001) and right ventricle global four-chamber longitudinal strain (RV4CSL) (*p* < 0.001), with the best measures reported in the control group and well-controlled asthmatic patients. There was a statistically significant difference between the studied groups regarding global longitudinal strain apical four-chamber (GLSA4C) (*p* = 0.007), global longitudinal strain apical two-chamber (GLSA2C) (*p* < 0.001), global longitudinal strain apical three-chamber (GLSA3C) (*p* < 0.001), and global longitudinal strain average (GLSAVG) (*p* < 0.001), with significantly better levels reported in the control group and well-controlled asthmatic patients.

**Figure 2:**
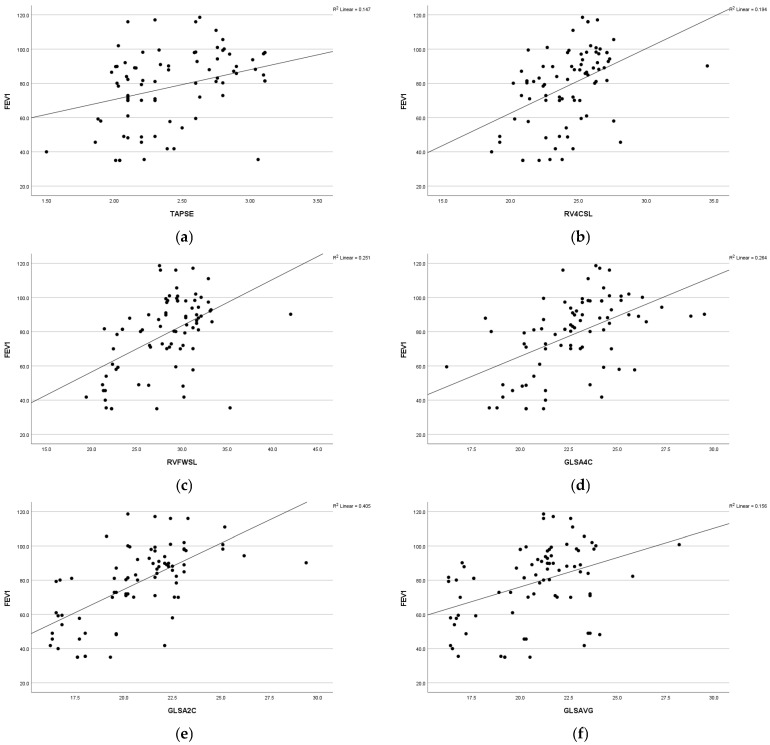
Scatter diagram illustrating the positive correlation between FEV1 and the following parameters: (**a**) TAPSE, (**b**) RV4CSL, (**c**) RVFWSL, (**d**) GLSA4C, (**e**) GLSA2C, and (**f**) GLSAVG among the studied groups.

A statistically significant positive association was observed between respiratory function (FEV1) and various parameters, including global longitudinal strain apical four-chamber view (GLS A4C) (r = 0.44 and *p* = 0.009), left ventricular global longitudinal strain apical two-chamber view (GLS A2C) (r = 0.58 and *p* =<0.001), left ventricular global longitudinal strain apical three-chamber view (GLS A3C) (r = 0.40 and *p* = 0.001), left ventricular global longitudinal strain average (GLS AVG) (r = 0.35 and *p* = 0.007), right ventricle free-wall longitudinal strain (RVFWSL) (r = 0.48 and *p* =< 0.001), and right ventricle four-chamber strain (RV4CSL) (r = 0.36 and *p* = 0.005). Furthermore, a significant positive association was found between respiratory function, forced expiratory volume in the first second (FEV1), and tricuspid annular plane systolic excursion (TAPSE) (r = 0.38 and *p* = 0.003). Statistically significant positive associations were observed among respiratory function, forced vital capacity (FVC), and the following parameters: GLSA2C (r = 0.43 and *p* =< 0.001), RV4CSL (r = 0.32 and *p* = 0.01), and RVFWSL (r = 0.39 and *p* = 0.002). Respiratory function (FVC) exhibited a positive correlation with fractional shortening (FS) (r = 0.34 and *p* = 0.008) (Figure 2).

**Primary outcome**

Table 3 shows that for TLR9 there was a statistically significant increase in the frequency of the CT and TT genotypes among asthmatic cases compared to the control group (OR = 9.09 and 5.24, respectively) and also in the T allele (OR = 3.23). TLR10 also showed a statistically significant increase in the frequency of the GT and TT genotypes among asthmatic cases compared to the control group (OR = 4.29 and 10.71, respectively) and also in the T allele (OR = 4.64).

**Secondary outcome**

The studied groups revealed statistically significant differences concerning TLR9 polymorphism, as the T allele and TT genotype were significantly detected among uncontrolled and partially controlled cases, while the C allele and CC genotype were equally detected in the normal control and well-controlled groups (Table 4).

Also, regarding TLR10 polymorphisms, the T allele and TT genotype were significantly detected among uncontrolled and partially controlled cases, while the G allele and GG genotype were significantly detected in the normal control and well-controlled groups, with no difference between the two groups (Table 4).

There was a statistically significant relation between the TLR9 gene polymorphisms and respiratory function FEV1% (*p* = 0.013) and FVC% (*p* = 0.004), as patients with the CC genotype had significantly better respiratory function (Table 5).

No significant statistical association was found between the TLR9 gene polymorphism and conventional cardiac functions, but there was a statistically significant relationship between the TLR9 gene polymorphism and both RVFWSL (*p* = 0.02) and GLS A2C (*p* = 0.024), with better values associated with the CC genotype (Table 5).

There was also a statistically significant relationship between the TLR10 gene polymorphism and respiratory function FEV1% (*p* = 0.047) and FVC% (*p* = 0.04), with the GG genotype having significantly better respiratory function than the TT genotype (Table 6).

There was no statistically significant relationship between the TLR10 gene polymorphism and conventional cardiac functions, and there was no statistically significant relationship between the TLR10 gene polymorphism and cardiac functions except GLS AVG (*p* = 0.042), which had a statistically significant relationship with the GG genotype and had significantly better cardiac function (Table 6).

## 4. Discussion

Asthma is a disease with many factors. The lack of consistent replication of genetic connections across different studies highlights the intricate interplay between various environmental and genetic aspects that contribute to the development of this condition [17]. Currently, ten functioning human TLRs (TLRs 1–10) have been identified, and their canonical adaptors’ pathways in a variety of human disorders, such as allergy diseases, are linked to them [18]. The new points of our study that have not been discussed in any study before are the relationship between TLR9 and TLR10 polymorphisms and subclinical cardiac dysfunction in asthmatic patients.

Our study revealed that both groups (asthmatics and controls) are closely matched as regards their age and sex with no significant statistical differences among the investigated groups (*p*-values were 0.321 and 0.656, respectively). This can be explained by the fact that the asthmatic cases were recruited first, and then another non-asthmatic, unrelated, non-allergic age- and gender-matched control group with similar ethnicity was randomly chosen later on. In our study, we found a distinction among asthmatic children and children of the control group concerning family history. Most of the asthmatic patients confirmed a familial predisposition to asthma.

Our findings align with previous studies conducted by Hernandez et al. [19], Yu et al. [20], and Guo et al. [21]. These studies reported no significant statistical differences among the groups under study as regards age and gender. However, in these studies, significant differences were found among the investigated groups in terms of family history.

Regarding anthropometric measures, our results showed no significant difference between asthmatic children and control children concerning body mass index (BMI) due to matching between the two groups. In line with our findings, Yalçın et al. [22] and Ardura-Garcia et al. [23] indicated no significant differences in BMI among the examined groups.

On the other hand, several studies have found a significant association between BMI and asthma. An Egyptian study conducted by Al-Qerem and Ling [24] on children aged 7–12 years showed a positive association between asthma and increased BMI, and comparable findings were documented by Nahhas et al. [25] in Saudi children.

The current study found a highly statistically significant difference regarding eosinophilic count, with all asthmatic groups exhibiting considerably higher values compared to the controls. This finding agreed with those results obtained by Hassane et al. [26], who found a highly statistically significant high eosinophilic count in asthmatic children compared to the control group.

In our study, by using conventional echocardiography for the assessment of cardiac function in asthmatic patients and the control group, we found a significant difference between them regarding TAPSE. This result agreed with the results obtained by Betül et al. [27] and Özkan et al. [28], who found a significant difference between asthmatics and the control group regarding TAPSE, with the highest mean values reported in the control group. On the other hand, no significant difference in TAPSE between asthmatics and the control group was reported by other studies [29,30]. This difference in results may be related to the difference in the duration and severity of asthma among the studied populations.

Moreover, our study revealed that there was a statistically significant difference between asthmatics and the control group regarding pulmonary artery systolic pressure (PASP), with PASP being higher in asthmatics. Karasu et al. [31], Manti et al. [32], and De-Paula et al. [33] reported similar results to our study regarding PASP as they found PASP higher in asthmatics. This can be explained by recurrent hypoxemia and hypercapnia related to different cytokines and mediators associated with chronic inflammation of the airways in patients with asthma that lead to pulmonary vasoconstriction and pulmonary hypertension development [12].

On the other hand, by using the speckle tracking technique, our study revealed that there was a statistically significant difference between the asthmatic group and the control group regarding RV4CSL and RVFWSL, with the best measures reported in the control group.

Moreover, there was a statistically significant positive correlation between respiratory function (FEV1 and FVC) and RV4CSL and RVFWSL. It was noted that with decreased pulmonary function, there is a decrease in global longitudinal and free-wall strain of the RT ventricle. These results coincide with those found by Tuleta et al. [34], who reported reduced longitudinal strain values of the right ventricle in the asthmatic group in comparison to the non-asthmatic controls. The same results were obtained by Baystal and Has [35], who conducted a study on adult asthmatic patients in comparison to a control group. In addition, other study confirmed a significant correlation between pulmonary function tests (FEV1, FVC) and RV strain parameters, with lower pulmonary function associated with decreased RVGLS and RVFWS [36].

On the other hand, the study Abdelmohsen et al. [29] performed on 30 children with mild to moderate asthma found no significant difference between the asthmatic group and the control group regarding right ventricle strain patterns. These differences can be attributed to a small sample size and the focus of their study being on patients with mild to moderate asthma.

Regarding LV systolic functions detected by conventional echocardiography, our study revealed that ejection fraction (EF) and fractional shortening (FS) were preserved in the asthmatic group. This coincides with the results obtained by Abdelmohsen et al. [29] and Ozdemir et al. [37] regarding EF.

Using speckle tracking echocardiography, we detected statistically significant differences between the four studied groups regarding left ventricle longitudinal strain, with the best measures reported in the control group. Our results were similar to those of Tuleta et al. [34], who found reduced LV longitudinal strain in patients with severe and mild-to-moderate asthma.

On the other hand, Bystal and Has [35] and Abdelmohsen et al. [29] reported no left ventricular stain pattern in asthmatic patients. This could be due to the fact that their study focused on patients with mild asthma. Moreover, we investigated TLR9 (rs 187084) and TLR10 (rs 11096956) gene polymorphisms and their association with asthma.

In our study, we found a statistically significant difference between the studied groups regarding TLR9 polymorphisms with the TT genotype and T allele in the uncontrolled (55%) and partially controlled cases (35%). The CC genotype and C allele were significantly detected in the normal control (67.5%) and well-controlled groups (55%), with no difference between the two groups. Our data showed a statistically significant relation between TLR9 gene polymorphisms and respiratory function, with the CC genotype having significantly better respiratory function. This coincides with the results of Tesse et al. [38], who reported an association between TLR9 (rs 187084) and asthma. Also, Kormann et al. [39] evaluated assumed functional genetic variants in all 10 human TLR genes, including TLR9, for their relation to different asthma phenotypes in a case-control study, which revealed that TLR9 (rs 187084) was associated with asthma with a *p*-value of 0.03.

Moreover, our data revealed a statistically significant relationship between the TLR10 gene polymorphisms and respiratory function, with the GG genotype having significantly better respiratory function than the TT genotype. This agrees with the results obtained by Tesse et al. [38], who found a significant association between TLR10 gene polymorphisms and asthma. However, Klaassen et al. [40] carried out a systematic review of TLRs and CD14 in relation to asthma in Caucasian children and found no association with bronchial asthma. Puthothu and Heinzmann [41], who involved 322 asthmatic children and 270 randomly selected controls to assess whether TLR6, TLR10, or both were involved in asthma genetics, found no individual association between TLR10 (rs11096956) and bronchial asthma.

The degree of association between polymorphisms and asthma varies between different populations and sometimes in the same population depending on environmental factors, genetics, sex differences, age of the studied patients, study methodologies, and differences in the number of subjects [11]. Asthma is a complex disease influenced by both genetic and environmental factors. If a population was exposed to different environmental triggers (e.g., allergens, pollution, infections), TLR10 (rs11096956) might play a role in asthma susceptibility in the presence of specific environmental conditions. Additionally, interactions with other genetic variants (epistasis) might modulate the effect of TLR10 (rs11096956), making its association apparent in our study but not in other studies.

This study aimed to investigate the association between TLR9 and TLR10 gene polymorphisms and bronchial asthma susceptibility, disease control, and their impact on respiratory and cardiac functions. TLR9 and TLR10 were linked to better asthma control and improved cardiac function, while subclinical left and right ventricular dysfunction was observed in asthmatic children. These findings suggest that TLR9 and TLR10 polymorphisms may serve as potential biomarkers for predicting asthma control and assessing the risk of asthma-related cardiac dysfunction. Our findings open avenues for several future research directions, e.g., large-scale genetic association studies conducting multi-center genome-wide association studies (GWAS), longitudinal cohort studies following asthmatic patients over time to assess how TLR9 and TLR10 polymorphisms influence disease progression, exacerbations, and long-term cardiac and pulmonary outcomes, and therapeutic targeting of the TLR pathways could improve asthma control and reduce associated cardiac dysfunction.

### Strengths and Limitations

This study represents the first attempt to examine the association between TLR gene polymorphisms, asthma control, and cardiac function in Egyptian children. It included several assessments (family history, clinical, laboratory, PFTs, and echo and gene polymorphism). The study results can be generalized due to the use of broad inclusion criteria that resulted in a study population that more closely resembles real-life patients, an adequate sample size, and an adjusted power and CI for the study.

It focused on TLR-9 and TLR-10 polymorphisms, prioritizing common variants due to resource limitations. Future research could expand to include rarer polymorphisms. Furthermore, although statistically adequate, the sample size could have been larger and had more depth for relationships within subgroups. Additionally, a more detailed evaluation of asthma treatments, patient history, and respiratory function is recommended (e.g., FEF 25%, FEF 50%, FEF 75%, FIF 50%, FEV/SVC, FEF 25–75%) as these factors were not comprehensively documented in the current study. In terms of cardiac parameters, future studies might benefit from employing more refined metrics (e.g., LVDs, LVDd, E velocity, A velocity, E/A).

## 5. Conclusions

The study aimed to verify the link between TLR9 and TLR10 gene polymorphisms and bronchial asthma and its control. The CC genotype of TLR9 and the GG genotype of TLR10 are associated with better asthma control. Also, we aimed to find a relationship between TLR9 and TLR10 gene polymorphisms and respiratory and cardiac functions. Subclinical cardiac dysfunction of the left and right ventricles was detected in asthmatic children. The CC genotype of TLR9 and the GG genotype of TLR10 are associated with better cardiac function. So, we conclude that studying TLR9 and TLR10 polymorphisms can be of value in predicting bronchial asthma control in asthmatic patients and cardiac dysfunction.

## Figures and Tables

**Table 1:** Demographic features of the four groups included in the study.

Variable	Uncontrolled	Partially Controlled	Well-Controlled	Control	Tests	
	Group	Group	Group	Group	*F*	*p*-Value
	(*n* = 20)	(*n* = 20)	(*n* = 20)	(*n* = 20)		
Age (years)						
Mean ± SD	8.15 ± 2.78	9.40 ± 4.08	7.75 ± 2.73	9.05 ± 2.82	1.184	0.321
Range	(5–13)	(5–15)	(5–14)	(5–14)		
Height (cm)						
Mean ± SD	133.55 ± 20.27	132.05 ± 18.96	126.25 ± 14.74	134.7 ± 17.67	0.867	0.462
Range	(106–168)	(105–165)	(106–155)	(106–165)		
Weight (kg)						
Mean ± SD	35.65 ± 14.12	34.30 ± 13.76	28.75 ± 10.43	32.50 ± 12.55	1.094	0.357
Range	(21–63)	(18–66)	(17–50)	(19–66)		
BMI (kg/m^2^)						
Mean ± SD	19.41 ± 4.27	19.12 ± 4.43	17.53 ± 3.19	17.34 ± 2.92	1.59	1.79
Range	(14.8–24)	(11.03–29.33)	(13.43–24.45)	(12.77 ± 24.4)		
Variable	N (%)	N (%)	N (%)	N (%)	χ^2^	*p*-value
Sex						
Female	11 (55)	9 (45)	7 (35)	9 (45)	1.616	0.656 (ns)
Male	9 (45)	11 (55)	13 (65)	11 (55)		
Family history						
Negative	5 (25)	1 (5)	3 (15)	20 (100)	48.627	<0.001 **
Positive	15 (75)	19 (95)	17 (85)	0		

N: number, SD: standard deviation; (*F)* one-way ANOVA, (χ^2^) chi-square test χ^2^; (**) highly significant. (ns) non significant.

**Table 2:** Mean right ventricular echocardiographic measurements and longitudinal strain pattern in speckle tracking in the four studied groups.

Variable	Uncontrolled Group (*n* = 20)	Partially Controlled (*n* = 20)	Well-Controlled (*n* = 20)	Control Group (*n* = 20)	Tests		
					*F*	*p*-Value	Post Hoc
Ejection Fraction EF% Mean ± SD Range	70.43 ± 3.95 (62–75.1)	70.08 ± 2.74 (64.3–74.3)	71.40 ± 3.67 (63–78.1)	71.54 ± 2.51 (66.6–77)	0.961	0.416	P1 = 0.740 P2 = 0.349 P3 = 0.287 P4 = 0.206 P5 = 0.164 P6 = 0.897
Fractional Shortening FS% Mean ± SD Range	36.3 ± 8.84 (2–44.1)	37.71 ± 2.93 (32.3–44.3)	39.06 ± 2.67 (32.6–43.2)	39.36 ± 2.58 (34.1–43.5)	1.570	0.204	P1 = 0.375 P2 = 0.085 P3 = 0.057 P4 = 0.397 P5 = 0.301 P6 = 0.850
Pulmonary Artery Systolic Pressure PASP (mm Hg) Mean ± SD Range	28.8 ± 7.35 (2–35)	27.5 ± 2.84 (24–33)	26 ± 3.76 (20–33)	25 ± 2.25 (22–29)	2.745	0.049 *	P1 = 0.365 P2 = 0.053 P3 = 0.009 * P4 = 0.296 P5 = 0.083 P6 = 0.485
TAPSE (cm) Mean ± SD Range	2.18 ± 0.34 (1.5–3.06)	2.39 ± 0.36 (2.02–3.11)	2.49 ± 0.38 (1.98–3.1)	2.74 ± 0.64 (2.02–3.07)	5.480	0.002 *	P1 = 0.133 P2 = 0.031 * P3 < 0.001 ** P4 = 0.449 P5 = 0.016 * P6 = 0.077
RV4CSL Mean ± SD Range	−22.94 ± 2.62 (−28.1)–(−18.6)	−23.26 ± 1.95 (−27.1)–(−20.2)	−25.33 ± 2.86 (−34.5)–(−20.8)	−25.49 ± 1.36 (−27.6)–(−2.4)	6.95	<0.001 *	P1 = 0.658 P2 = 0.001 * P3 = 0.001 * P4 = 0.005 * P5 = 0.003 * P6 = 0.825
RVFWSL Mean ± SD Range	−24.89 ± 4.32 (−35.3)–(−19.4)	−27.28 ± 3.02 (−31.8)–(−21.4)	−30.71 ± 3.47 (−42)–(−24.2)	−30.2 ± 2.05 (−33.3)–(−26.3)	13.36	<0.001 *	P1 = 0.025 * P2 < 0.001 ** P3 < 0.001 ** P4 = 0.002 * P5 = 0.007 * P6 = 0.628
GLS A4C Mean ± SD Range	−18.95 ± 9.79 (−25.9)–(−21.3)	−22.08 ± 1.68 (−25.1)–(−18.5)	−23.78 ± 2.62 (−29.5)–(−26.5)	−24.31 ± 1.19 (−26.5)–(−22.2)	4.360	0.007 *	P1 = 0.059 P2 = 0.0048 * P3 = 0.002 * P4 = 0.302 P5 = 0.178 P6 = 0.749
GLS A2C Mean ± SD Range	−18.12 ± 1.88 (−22.5)–(−16.2)	−20.29 ± 1.98 (−24.1)–(−16.5)	−22.31 ± 2.14 (−29.4)–(−19.6)	−22.69 ± 1.85 (−26.7)–(−19.1)	22.8	<0.001 *	P1 = 0.001 * P2 < 0.001 * P3 < 0.001 * P4 = 0.002 * P5 < 0.001 * P6 = 0.548
GLS A3C Mean ± SD Range	−18.37 ± 1.42 (−21.3)–(-16.2)	−19.5 ± 1.91 (−22.8)–(-16.1)	−19.83 ± 1.6 (−24.1)–(−16.9)	−20.83 ± 1.35 (−22.9)–(−18.6)	8.14	<0.001 *	P1 = 0.027 * P2 = 0.005 * P3 < 0.001 ** P4 = 0.519 P5 = 0.010 * P6 = 0.050
GLS AVG Mean ± SD Range	−16.98 ± 9.24 (−24.1)–20	20.12 ± 2.53 (−24)–(−16.3)	21.35 ± 1.99 (−25.8)–(−17)	22.61 ± 1.67 (−28.2)–(−20)	4.72	<0.001 *	P1 = 0.049 * P2 = 0.007 * P3 = 0.001 * P4 = 0.436 P5 = 0.117 P6 = 0.425

(F) one-way ANOVA; (*) significant (<0.05), (**) highly significant (<0.001); P1 = uncontrolled group vs. partially controlled group, P2 = uncontrolled group vs. well-controlled group, P3 = uncontrolled group vs. control group, P4 = partially controlled group vs. well-controlled group, P5 = partially controlled group vs. control group, P6 = well-controlled group vs. control group.

**Table 3:** TLR9 and TLR10 gene polymorphisms among the studied groups.

Variable	Cases		Control		*p*	χ^2^	OR
	(*n* = 60)		(*n* = 20)				
	N	%	N	%			
TLR9:							
CC	11	18.3	12	60	--	--	Reference
CT	25	41.7	3	15	10.45	0.001 *	9.09 (2.13–38.77)
TT	24	40	5	25	7.11	0.008 *	5.24 (1.48–18.53)
Allele:							
C	47	39.2	27	67.5	9.69	0.002 *	3.23 (1.51–6.87)
T	73	60.8	13	32.5			
TLR10:							
GG	21	35	15	75	--	---	Reference
GT	24	40	4	20	5.66	0.02 *	4.29 (1.23–14.94)
TT	15	25	1	5	6.52	0.01 *	10.71 (1.27–90.14)
Allele:							
G	66	55	34	85	11.52	<0.001	4.64 (1.81–11.86)
T	54	45	6	15		**	

N: number, χ^2^: chi square test, OR: odds ratio; *: significant (*p* < 0.05), **: highly significant (*p* < 0.001).

**Table 4:** TLR9 and TLR10 gene polymorphism distribution in the studied groups.

Variable	Uncontrolled Group (*n* = 20)		Partially Controlled Group (*n* = 20)		Well-Controlled Group (*n* = 20)		Control Group (*n* = 20)		Tests		Multi Comparison Analysis
									χ^2^	*p*-Value	
	N	%	N	%	N	%	N	%			
TLR9 polymorphism											
CC (n = 23)	0	0	3	15	8	40	12	60	21.887	0.001 *	P1 = 0.139 P2 = 0.007 * P3 < 0.001 ** P4 = 0.187 P5 = 0.009 * P6 = 0.389
TC (n = 28)	9	45	10	50	6	30	3	15			
TT (n = 29)	11	55	7	35	6	30	5	25			
TLR9 Allele											
C (n = 74)	9	22.5	16	40.0	22	55.0	27	67.5	18.2	0.001 *	P1 = 0.09 P2 = 0.002 * P3 < 0.001 ** P4 = 0.178 P5 = 0.013 * P6 = 0.251
T (n = 86)	31	77.5	24	60.0	18	45.0	13	32.5			
TLR10 polymorphism											
GG (n = 36)	4	20	7	35	10	50	15	75	18.8	0.004 *	P1 = 0.557 P2 = 0.018 * P3 = 0.001 ** P4 = 0.114 P5 = 0.026 * P6 = 0.232
GT (n = 28)	8	40	7	35	9	45	4	20			
TT (n = 16)	8	40	6	30	1	5	1	5			
TLR10 Allele											
G (n = 100)	16	40	21	52.5	29	72.5	34	85	20.6	0.001 **	P1 = 0.262 P2 = 0.003 * P3 = 0.001 ** P4 = 0.06 P5 = 0.001 ** P6 = 0.171
T (n = 60)	24	60	19	47.5	11	27.5	6	15			

N: number, χ^2^: chi square test; (*) significant, (**) highly significant; P1 = uncontrolled group vs. partially controlled group, P2 = uncontrolled group vs. well-controlled group, P3 = uncontrolled group vs. control group, P4 = partially controlled group vs. well-controlled group, P5 = partially controlled group vs. control group, P6 = well-controlled group vs. control group; CC: homozygous wild type, TT: homozygous mutant type, TC: heterozygous mutant type.

**Table 5:** Comparison between TLR9 gene polymorphisms and respiratory and cardiac functions and ECHO findings within the studied groups.

Variable	TLR9			Tests		
	CC	CT	TT	*F*	*p*-Value	Post Hoc
Respiratory function						
FEV1%						P1 = 0.006 *
Mean ± SD	85.72 ± 6.42	67.32 ± 19.62	67.59 ± 19.29	4.694	0.013 *	P2 = 0.007 *
Range	(71–94.3)	(35–97.1)	(35–99.5)			P3 = 0.958
FVC%						P1 = 0.244
Mean ± SD	83.02 ± 11.99	76.5 ± 14.62	71.18 ± 17.19	2.337		P2 = 0.038 *
Range	(60.2–97.4)	(52–104)	(29.5–96)		0.0.04 *	P3 = 0.229
Cardiac function						
Ejection Fraction EF%						P1 = 0.083
Mean ± SD	72.41 ± 2.87	70.22 ± 3	70.26 ± 4.03	1.801	0.174	P2 = 0.091
Range	(69.1–78.1)	(63–75.1)	(62–77.4)			P3 = 0.966
Fractional Shortening FS%						P1 = 0.424
Mean ± SD	37.59 ± 3.06	39.2 ± 2.73	36.15 ± 8	1.861	0.165	P2 = 0.479
Range	(32.6–42.7)	(32.3–44.3)	(2–44.1)			P3 = 0.059
Pulmonary Artery Systolic Pressure PASP (mm Hg)						P1 = 0.496
Mean ± SD	26.36 ± 3.8	27.64 ± 3.76	27.71 ± 2	0.292	0.748	P2 = 0.476
Range	(20–31)	(23–35)	(2–34)			P3 = 0.963
TAPSE (cm)						P1 = 0.853
Mean ± SD	2.39 ± 0.35	2.36 ± 0.4	2.33 ± 0.38	0.091	0.913	P2 = 0.686
Range	(1.98–2.88)	(1.8–3.11)	(1.5–3.1)			P3 = 0.778
RV4CSL						P1 = 0.328
Mean ± SD	−24.59 ± 2.37	−23.62 ± 1.92	−23.73 ± 3.45	0.523	0.596	P2 = 0.386
Range	(−27.3)–(−20.8)	(−27.6)–(−20.8)	(−34.5)–(−18.6)			P3 = 0.892
RVFWSL						P1 = 0.230
Mean ± SD	−30.01 ± 2.5	−28.21 ± 3.25	−25.92 ± 5.29	4.183	0.02 *	P2 = 0.008^*^
Range	(−33.1)–(−25.6)	(−35.3)–(−22.2)	(−42)–(−19.4)			P3 = 0.056
GLS A4C						P1 = 0.456
Mean ± SD	−23.7 ± 2.72	−22.04 ± 2.06	−20.19 ± 9.25	1.341	0.270	P2 = 0.122
Range	(−28.8)–(−20.3)	(−25.1)–(−16.2)	(−29.5)–(−21.3)			P3 = 0.297
GLS A2C						P1 = 0.271
Mean ± SD	−21.62 ± 1.91	−20.62 ± 1.68	−19.22 ± 3.3	3.983	0.024 *	P2 = 0.011 *
Range	(−26.2)–(-19.5)	(−22.8)–(−16.8)	(−29.4)–(−16.2)			P3 = 0.055
GLS A3C		−19.15 ± 1.39				P1 = 0.337
Mean ± SD	−19.76 ± 1.94	(−22.5)–(-17.1)	−19.08 ± 2	0.629	0.537	P2 = 0.286
Range	(−24.1)–(−16.9)		(−22.8)–(−16.1)			P3 = 0.885
GLS AVG						P1 = 0.256
Mean ± SD	−21.46 ± 1.79	−19.02 ± 8.58	−19.05 ± 2.71	0.768	0.469	P2 = 0.263
Range	(−23.6)–(−17.6)	(−25.8)–(−20.5)	(−24)–(−16.3)			P3 = 0.990

SD: standard deviation; (*F*) one-way ANOVA, (ns) non-significant, (*) significant; P1 CC vs. CT, P2 CC vs. TT, P3 CT vs. TT.

**Table 6:** Comparison between the TLR10 gene polymorphisms and respiratory and cardiac functions and ECHO findings within the studied groups.

Variable	TLR10			Tests		
	CC	CT	TT	*F*	*p*-Value	Post Hoc
Respiratory function						
FEV1%						P1 = 0.220
Mean ± SD	77.05 ± 16.57	70.2 ± 19.73	63.01 ± 18.94	2.544	0.047 *	P2 = 0.029 *
Range	(41.8–97.1)	(35–99.5)	(35–90.2)			P3 = 0.242
FVC%						P1 = 0.361
Mean ± SD	80.38 ± 15.14	76.22 ± 13.46	67.77 ± 17.52	3.076	0.04 *	P2 = 0.017 *
Range	(43–104)	(52–97.4)	(29.5–86.8)			P3 = 0.095
Cardiac function						
Ejection Fraction EF%						P1 = 0.368
Mean ± SD	70.37 ± 3.34	71.31 ± 3.09	69.93 ± 4.23	0.824	0.444	P2 = 0.710
Range	(63–78.1)	(64.3–75.5)	(62–77.4)			P3 = 0.232
Fractional Shortening FS%						P1 = 0.498
Mean ± SD	37.98 ± 3.26	39.09 ± 2.6	35.04 ± 9.77	2.576	0.085	P2 = 0.118
Range	(32.3–43.2)	(34.9–44.3)	(2–42.1)			P3 = 0.498
Pulmonary Artery Systolic Pressure PASP (mm Hg)						P1 = 0.982
Mean ± SD	27.29 ± 3.74	27.25 ± 4.11	27.93 ± 7.81	0.094	0.911	P2 = 0.712
Range	(21–35)	(20–34)	(2–34)			P3 = 0.689
TAPSE (cm)						P1 = 0.148
Mean ± SD	2.45 ± 0.38	2.29 ± 0.36	2.31 ± 0.39	1.198	0.309	P2 = 0.265
Range	(2.01–3.11)	(1.86–3.06)	(1.5–3.1)			P3 = 0.861
RV4CSL						P1 = 0.726
Mean ± SD	−23.82 ± 2.24	23.53 ± 2.36	−24.37 ± 3.69	0.444	0.643	P2 = 0.548
Range	(−27.2)–(−19.2)	(−28.1)–(−19.2)	(−34.5)–(−18.6)			P3 = 0.351
RVFWSL						P1 = 0.775
Mean ± SD	−28.24 ± 3.33	−27.87 ± 3.85	−26.37 ± 6	0.876	0.422	P2 = 0.207
Range	(−33.2)–(−21.2)	(−35.3)–(−21.3)	(−42)–(−19.4)			P3 = 0.299
GLS A4C						P1 = 0.159
Mean ± SD	−22.82 ± 2.06	−20.2 ± 9.14	−22.15 ± 3.34	1.096	0.341	P2 = 0.750
Range	(−28.8)–(−19.1)	(27.3-)–(−21.3)	(−29.5)–(−16.2)			P3 = 0.338
GLS A2C						P1 = 0.358
Mean ± SD	−20.86 ± 1.67	−20.13 ± 2.52	−19.55 ± 3.65	1.135	0.328	P2 = 0.143
Range	(−23.1)–(−16.3)	(−26.2)–(−16.3)	(−29.4)–(−16.2)			P3 = 0.498
GLS A3C	−19.21 ± 1.1	−19.58 ± 1.9	−18.71 ± 2.16			P1 = 0.485
Mean ± SD	(−21.6)–(−16.9)	(−24.1)–(−17.1)	(−22.8)–(−16.1)	1.133	0.329	P2 = 0.403
Range						P3 = 0.138
GLS AVG	−21.98 ± 1.82	−18.55 ± 8.53	−17.47 ± 2.22			P1 = 0.047 *
Mean ± SD	(−25.8)–(−17.6)	(−23.5)–(−20.5)	(−24)–(−16.2)	3.344	0.042(S)	P2 = 0.021 *
Range						P3 = 0.560

SD: standard deviation, (*F*) one-way ANOVA, (*) significant; P1 CC vs. CT, P2 CC vs. TT, P3 CT vs. TT.

## Data Availability

The datasets used and analyzed during the current study are available from the corresponding author on reasonable request.
